# Analysis of the prevalence and related factors of primary hypertension among adolescents and children in the Taicang area

**DOI:** 10.1186/s12887-023-04061-7

**Published:** 2023-05-26

**Authors:** Zhongxing Lu, Yan Teng, Lifang Wang, Lishan Jia, Zhiyun Chen, Shouling Ding

**Affiliations:** 1 Department of Pediatrics, Changzhou maternal and Child Health Hospital Affiliated to Nanjing Medical University, Nanjing, China; 2 Department of Pediatrics, Taicang Affiliated Hospital of Soochow University, Suzhou, 215400 Jiangsu Province China; 3 Department of Pediatrics, Taicang Affiliated Hospital of Soochow University, No. 58 Changsheng South Road, 215400, 215400 Taicang, Jiangsu Province China

**Keywords:** Primary hypertension, Prevalence, Children and adolescents, Influencing factors

## Abstract

**Objective:**

To investigate the prevalence of hypertension in adolescents and children in the Taicang area and analyse related factors to provide a theoretical basis for the prevention and control of hypertension in this region.

**Methods:**

A total of 1,000 students who were visited and surveyed in primary schools in the Taicang area in 2021 were selected for statistical testing using a cluster random sampling method, and a survey was conducted on their dietary habits. dietary habits, such as the consumption of meals that included protein-rich animal products, beans and dairy products, vegetables and fruits, salty foods and fried food, was taken into consideration, along with physical fitness indices, waist-to-height ratio and waist circumference.

**Results:**

Of the 1,000 adolescents and children surveyed, 222 were classified into the hypertensive group and 778 into the normotensive group. There were 138 boys (a prevalence rate of 6.3%) and 84 girls (a prevalence rate of 4.1%) in the hypertensive group. The physical fitness indices of the hypertensive group were significantly higher than those of the normotensive group. Concerning dietary structure, the frequency of cereal intake between the two groups was comparable, while the hypertensive group’s intake of vegetables, fruits, beans and dairy products was significantly lower than that of the normotensive group. Finally, a logistic multivariate regression analysis of related factors was carried out, and it was concluded that waist-to-height ratio, waist circumference and salty and fried food intake were positively correlated with the prevalence of hypertension.

**Conclusion:**

The prevalence of hypertension among adolescents and children in the Taicang area is high. Body weight and dietary structure can be used as reference indicators for the prevalence of hypertension in this age group.

## Introduction

Hypertension is a chronic disease characterised by a continuous increase in arterial blood pressure (BP). Hypertension is classified as primary or secondary, depending on its different causes. Primary hypertension is the most common type of hypertension in adolescents and children, and relevant data show that nearly 2% of adolescents and children suffer from primary hypertension. However, hypertension in children and adolescents causes fewer complications, and its mechanism has been relatively well-studied to date [1–3]. Globally, approximately 1 billion people over the age of 25 have been diagnosed with hypertension. Known as the ‘silent killer’, hypertension is a major risk factor for cardiovascular and cerebrovascular diseases. [4–6] Recent studies have shown that BP in adolescents and children is closely associated with subsequent adult BP; thus, increasing attention is being paid to the prevention and treatment of hypertension in adolescents and children [7]. As China’s socioeconomic conditions and living standards continue to improve, rates of hypertension are also rising, with younger populations increasingly affected [8]. From 1991 to 2004, it was found that the prevalence of hypertension among children and adolescents in seven cities and provinces throughout China increased from 7.1 to 14.6%, a trend that is increasing annually [9]. A 2010 survey of inhabitants of Beijing found the prevalence of hypertension among adolescents and children to be persistently high, at a discouraging 8.9%. For this reason, with the support of the 12th Five-Year National Science and Technology Support Program, the fifth survey to measure data on hypertension and related disease statistics was carried out in China, and for the first time, adolescents and children were included among the surveyed populations. This action contributes to the comprehensive understanding of the prevalence and risk factors of hypertension, helping relevant departments to formulate more reasonable policies and protective measures [10].

Many factors affect the prevalence of hypertension in adolescents. For example, inheritance influences the occurrence of hypertension under changing sodium levels and water reabsorption. A high-sodium, low-potassium diet is another important risk factor for teenage hypertension, as the risk of hypertension shows a positive correlation with an increase in sodium intake [11]. Moreover, since hypertension is a psychosomatic disease, factors such as living environment, personality, psychological state and behavioural issues are closely associated with BP [12]. However, the interactions of these influencing factors, as well as how each affects hypertension, are not clearly understood. To better prevent and raise awareness around hypertension and understand its prevalence and related risk factors in adolescents and children, we surveyed 1,000 children and adolescents aged 6–16 years in the Taicang area.

## Data and methods

### General information

A whole-group, random sampling method was adopted for this cohort study. Ten per cent of the overall population of 10,000 elementary school students in the Taicang area in 2021 was sampled, with a total sample size of 1,000 students. Schools were divided into urban elementary and township elementary schools, according to the areas in which they were located. One urban elementary school was randomly selected, and the students of that school were enrolled as subjects of this study. If the number of students at the elementary school exceeded 500, only 500 were surveyed; if the number of students was less than 500, a second elementary school was randomly selected until 500 students were drawn. According to the same method, an additional 500 students in township elementary schools were randomly selected. This study was conducted following the Declaration of Helsinki and approved by the Ethics Committee of the Taicang First People’s Hospital (approval code: KY-2022-356). Consent was obtained from the parents or guardians of all study subjects.

### Methods

Blood pressure, weight, height, waist circumference and waist-to-height ratio were collected for all study subjects, and a questionnaire assessing the frequency of consumption of each food group (i.e. grains, foods rich in animal protein, beans, dairy products, vegetables and fruits, and salty and fried foods) was conducted.

Blood pressure was measured using an electronic sphygmomanometer, and the adolescent or child was instructed to maintain an empty stomach for 1 h prior to taking the measurement and to avoid strenuous exercise, food and drink (except water). Blood pressure was measured by an internationally accepted method, and the size of the BP cuff varied according to the length and thickness of the upper arm of the adolescent or child, with the width of the balloon inside the cuff being two-thirds that of the upper arm (not including ends or overlap). The measurement was performed with the study subject sitting in a back-supporting chair with the body straight and the centre of the cuff at the same level as the heart. The right, upper extremity was measured for systolic (K1) and diastolic (K5) BP at intervals of 1–2 min each time, with an average of three or more measurements taken as the result. The second and third measurements were taken at intervals of about one week if the first BP reading was high, and hypertension was registered if three consecutive high BP readings occurred. The diagnostic criteria for BP were adopted from the 2010 Chinese Guidelines for the Prevention of Hypertension. High BP was assessed as a systolic/diastolic BP at or above the 95th percentile for children of the same height, sex and age at three or more consecutive readings; normal BP was deemed a systolic/diastolic BP below the 95th percentile for children of the same sex, age and height.

Simultaneously, a comprehensive health examination of the study subjects was conducted. The physical examination included height, weight, waist circumference and waist-to-height ratio, and the diagnostic criteria for obesity followed the classification standards developed in 2004 for screening Chinese school-aged children and adolescents for overweightness and obesity using body mass index (BMI) values [13].

After parents and guardians signed informed consent forms, the frequency of the study subjects’ consumption of the aforementioned foods in the past 12 months was assessed by questionnaire. If the frequency of intake was less than once a day, it was recorded as ‘several times a week’; if the frequency of intake was less than once a week, it was recorded as ‘several times a month’. Diets consisted mainly of coarse grains (e.g. maize, wheat, purple rice, sorghum), fine grains (e.g. refined rice, white flour and its products), meat (e.g. pork, beef, mutton, poultry), aquatic products (e.g. fish, shrimp, shellfish), eggs, milk and dairy products (e.g. fresh milk, powdered milk, cheese), pickled products (e.g. pickled vegetables, meat and eggs), salty foods (e.g. chips, biscuits), sugary foods (jams, cakes, chocolate, candy, desserts, colas, juices, etc.), sugar (e.g. white and brown sugar, honey, syrup), bean products (e.g. soy milk, tofu, bean sprouts, peas, beans), nuts (walnuts, almonds, chestnuts, melon seeds, peanuts, etc.), fruits and pure juices, leafy vegetables (spinach, cabbage, etc.), non-leafy, cooked vegetables, etc. These foods were classified into cereals, foods rich in animal protein, beans and dairy products, vegetables and fruits, salty foods and fried foods.

Physical exercise: A questionnaire was designed to investigate and record the typical physical exercise of all study subjects (in *n* hours/week). Results were divided into three levels: <1 h/week, 1–2 h/week and ≥ 2 h/week.

### Inclusion and exclusion criteria

After the questionnaires were collected, the study supervisor was responsible for their review and entry, and any invalid’ of unqualified questionnaires were returned to the survey to ensure the credibility of the questionnaires to the greatest degree possible.

### Statistical methods

The SPSS v. 23.0 statistical software was used for data sample analysis and continuous data were expressed as mean ± standard deviation. The differences between group and intra-group means were analysed by the *t*-test method, and categorical variables were analysed with the chi-squared (χ^2^) test. Risk factors were identified via logistic analysis and the results were considered statistically significant at *P* < 0.05.

## Results

### Basic characteristics of study participants

The 570 boys and 430 girls surveyed were divided into 10 groups, according to age (6–15 years); 143 students were aged 6 years, 179 aged 7, 190 aged 8, 168 aged 9, 137 aged 10, 104 aged 11, 47 aged 12, 19 aged 13, 9 aged 14 and 4 aged 15.

### Morbidity in the hypertensive group

Among the 1,000 adolescents and children in the Taicang area who met the study criteria, 222 were classified into the hypertensive group and 778 into the normotensive group. Within the hypertensive group, there were 138 boys and 84 girls with a prevalence rate of 6.3% and 4.1%, respectively. The prevalence rate among boys was significantly higher than that among girls.

### Comparison of physical indicators between normo- and hypertensive groups

Data on physical fitness indicators, including height, weight, BMI, waist circumference and waist-to-height ratio, were collected for all study participants, and an independent sample *t*-test was used to analyse results, as shown in Table 1. The physical fitness indicators of the hypertensive group were all significantly higher than those of the normotensive group (see Table 1).

**Table 1:** Results of the analysis of physical indicators and blood pressure in the normal and hypertensive groups

Group	Height (cm)	Body weight (kg)	BMI (kg/m^2^ )	Waist circumference (cm)	Waist-to-Height Ratio	Systolic blood pressure (mmHg)	Diastolic blood pressure (mmHg)
Hypertensive group	137.23 ± 12.32	37.35 ± 10.24	19.52 ± 3.37	91.48 ± 6.01	0.67 ± 0.74	127.82 ± 8.44	71.31 ± 9.66
Normal group	127.45 ± 10.22	28.14 ± 7.22	17.08 ± 2.4	66.13 ± 4.52	0.52 ± 0.56	106.49 ± 8.58	62.6 ± 5.78
t	11.99	15.16	12.06	68.11	32.52	32.7	16.73
P	0.043	0.025	0.032	0.001	0.047	0.043	0.025

Variables were expressed as mean ± standard deviation. BMI: body mass index

### Relationship between BP and dietary habits

The relationship between BP and the frequency of consumption of various food groups is shown in Table 2. The food groups consumed by the study participants include cereals (i.e. coarse and fine grains), foods rich in animal protein (e.g. meat and eggs), beans and dairy products (i.e. bean products, nuts, milk and other dairy products), vegetables and fruits (e.g. fruits, pure juices, leafy and non-leafy vegetables), salty foods (e.g. soy sauce/other sauce products, pickled products, salty snacks) and fried foods. From the analysis in Table 2, it can be seen that the consumption of cereals was almost equal between groups, while the consumption of vegetables, fruits, beans and dairy products in the hypertensive group was significantly lower than in the normotensive group. Additionally, the consumption of protein-rich animal products and salty and fried foods was much higher in the hypertensive group than in the normotensive group.

**Table 2:** The relationship between blood pressure and the frequency of weekly food intake of adolescents

Group	Cereals	Animal protein-rich foods	Beans and Dairy Products	Vegetables and Fruits	Salty food	Fried food
Hypertensive group	15.27 ± 1.46	10.24 ± 0.95	6.61 ± 0.45	10.74 ± 1.58	11.73 ± 1.29	5.36 ± 0.9
Normal group	15.26 ± 0.93	7.66 ± 0.81	7.92 ± 0.68	14.54 ± 0.76	8.49 ± 1.03	3.5 ± 0.46
t	0.11	40.22	-26.78	-49.71	39.01	41.66
P	P = 0.92	0.031	0.025	0.015	0.033	0.045

Variables were expressed as mean ± standard deviation

### Relationship between BP and physical exercise

The relationships between BP and the frequency of physical exercise are shown in Table 3. According to the analysis in Table 3, the frequency of physical exercise per week was negatively associated with the risk of hypertension.

**Table 3:** Relationship between blood pressure and physical exercise frequency of adolescents

group	< 1 h/week(n/%)	1 ~ 2 h/week(n/%)	≥ 2 h/week(n/%)
Overweight group	98/44.14^*^	104/46.85^*^	20/9.01^*^
normal weight group	78/10.03	106/13.62	594/76.35

Variables were expressed as counts/percentage. Note: Compared with the normal weight group, *P < 0.05

### Morbidity in adolescents and children of different weights

The classification criteria for obesity among different age groups within the study were determined according to the overweight and obesity screening BMI values for Chinese school-aged children and adolescents formulated in 2004. One hundred thirty-five study participants were classified as obese, 98 as overweight and 767 as within the normal weight range. The prevalence of hypertension within these three groups was 21.3%, 5.62% and 1.82%, respectively (see Table 4).

**Table 4:** Comparison of Prevalence of Hypertension in Children with Different Weights

	Total number of participants	Participants with hypertension	Prevalence (%)	OR value(95%CI)
Obese group	135	29	21.3*	12.14(10.63 ~ 16.98)
Overweight group	98	6	5.62*	1.76(1.03 ~ 2.98)
normal weight group	767	14	1.82	0.85(0.63 ~ 1.74)

OR: odds ratio. Note: Compared with the normal weight group, *P < 0.01

### Analysis of associated factors of hypertension in children and adolescents

Regression analyses were performed on the high-risk factors affecting the prevalence of hypertension, based on the contents of the questionnaires and the results of the physical examinations. Hypertension prevalence was set as the dependent variable, and height, weight, BMI, waist circumference, waist-to-height ratio, cereals, protein-rich animal products, beans and dairy products, vegetables and fruits, salty foods and fried foods were set as covariates for multifactorial logistic analysis. After excluding the positive associations between hypertension and height, weight, BMI and frequency of physical exercise by stepwise logistic regression analysis (*P* for in = 0.05, *P* for out = 0.01), the factors ultimately selected for the multi-element regression equation were: waist circumference, waist-to-height ratio and frequency of consumption of protein-rich animal products, beans and dairy products, vegetables and fruits, salty foods and fried foods. Factors that had a significant effect on hypertension were waist-to-height ratio, waist circumference, the frequency of consumption of salty and fried foods (with a positive association) and the frequency of consumption of vegetables, fruits, beans and dairy products (with a negative association). Results are shown in Table 5.

**Table 5:** Logistic regression analysis of multi-factors affecting the prevalence of hypertension in children

Related factors	OR	95%CI	P
Waist-to-Height Ratio	4.31	3.16 ~ 4.89	<0.001
Waist Circumference	3.75	3.12 ~ 4.13	<0.001
Foods rich in plant and animal protein	2.75	1.95 ~ 3.34	0.85
Beans and Dairy Products	0.91	0.85 ~ 0.96	<0.001
Vegetables and Fruits	0.76	0.69 ~ 0.82	<0.001
Salty food	2.35	1.86 ~ 2.75	0.76
Fried food	2.57	1.91 ~ 2.93	0.63

OR: odds ratio

## Discussion

With improvements in socioeconomic conditions and quality of life, the prevalence of hypertension is trending upward and affecting younger groups [14]. Relevant studies have shown hypertension, which starts in childhood, is the main risk factor for severe cardiovascular and cerebrovascular events. Behavioural risk factors are formed in large part during childhood; therefore, preventing hypertension in adolescents and children is the most effective means of staving off these life-threatening events [15]. In this paper, we found that the prevalence of hypertension among adolescents and children in the Taicang area was persistently high, and the diastolic and systolic BPs of boys were significantly higher than those of girls, which is consistent with the findings of Dong et al. [16].

A large body of research has confirmed that obesity is a major risk factor for hypertension, and BP in adolescents and children is positively correlated with height, weight and BMI. [17, 18] Van Emmerik et al. studied the association between obesity and hypertension and found that, for every 10 kg increase in body weight, systolic and diastolic BPs increased by 3.0 mmHg and 2.3 mmHg, respectively [19]. Savva et al. showed that there is a significant association between obesity and the prevalence of hypertension, which has a predictive value for cardiovascular disease, and that effective interventions should be taken as early as possible to prevent and manage the occurrence of cardiovascular disease [20].

Poor dietary habits are another important factor contributing to the occurrence of hypertension. [21, 22] The results of related studies show that the frequency of consumption of fruits and fresh, green vegetables is negatively associated with the prevalence of hypertension. However, with an improvement in living standards, people are more inclined to consume high-calorie, high-protein and salty snacks, which can easily result in hypertension. In this study, the frequency of consumption of vegetables and fruits was significantly greater in the normotensive group than in the hypertensive group, and the weekly intake of foods rich in animal and soy products, salty foods, sweets and fried foods was significantly greater in the hypertensive group than in the normotensive group. There was virtually no difference between the two groups concerning the consumption of cereals. This may be due to a parental attitude of ‘controlling daily intake’. The current study suggests that the frequency of consumption of high-calorie foods, such as sweets and fried foods, may be positively correlated with BP and that controlling the intake of such foods, in general, can help control BP.

Waist circumference and waist-to-height ratios were significantly higher in the hypertensive group than in the normotensive group. The relationship between obesity and the prevalence of hypertension in adults has been demonstrated in relevant studies [23], and waist circumference is likewise a risk factor for hypertension in younger populations. Waist-to-height ratio, a derived index of waist circumference, has a relatively small degree of variability over a wider range and, thus, has the advantage of being relatively stable. The current study suggests that waist circumference and waist-to-height ratio can be relevant predictive factors for the prevalence of hypertension in children and adolescents.

Although SPSS v. 23.0 software was used in this study to conduct a preliminary analysis of the prevalence of hypertension, obesity and related influencing factors in the study population, a small sample size and regional limitations warrant further study of additional influencing factors.

This study also includes numerous limitations. For example, hypertension in teenagers is not well-defined. Additionally, no follow-up was conducted. The process of hypertension is much more important and will be discussed in future studies. When teenagers consume large amounts of high-fat food, their demand for carbohydrates is relatively reduced. In addition, although children enjoy sweet foods, the survey found that most parents are ‘controlling the quantity’ of these foods that their children consume; therefore, their total intake is not much related, and the specific reasons need to be further discussed.

## Conclusion

In summary, the prevalence of hypertension among adolescents and children in Taicang is high. Waist-to-height ratio, waist circumference, vegetable and fruit consumption and the intake of unhealthy foods are the main influencing factors for adolescent and childhood hypertension.

## Data Availability

All data generated or analyzed during this study are included in this published article.
